# Co-Operation and Charity

**Published:** 1897-05-08

**Authors:** 


### Co-operation and Charity.

The resolution arrived at last week by the Keighley co-operators to present 1,000 guineas to the building and endowment fund of the Keighley and District Hospital, not only shows how large is the scale on which co-operative industry is organised in some parts of the country, but also, we hope, may be taken as an illustration of the fact that the great co-operative societies are willing to take upon themselves a share of those works of charity which have gradually become an inseparable part of our social system.

At the quarterly meeting of the Keighley Co-operative Society, Mr. Frank Midgley, the president, among the reasons which he gave for their making this gift said that by co-operation they had displaced a lot of people who would otherwise have been subscribers to an institution of this kind.

This seems to us to show a grasp of the true meaning and of the ulterior consequences of recent economic changes in the world of industry which we could wish was more generally felt, not only by co-operators, but by shareholders in great manufacturing and commercial concerns. With the spread of co-operation and the extension of the limited liability principle there is a great risk of the personal responsibility of the "master" to his workpeople being lessened. The employer of labour is a board instead of an individual, and the more completely the two classes of employer and employed are merged in one, as is the case where co-operative industry is at work, the more must that independent class diminish which has hitherto been the backbone of our charitable institutions.

We must, then, thank the co-operators of Keighley for reminding the world that when new forms of industry displace old classes of givers they must become givers themselves; and we are glad to find that in this instance the gift is to be a real one! If they will add to the charity of their free gift the equally important charity of personal service in the management of the hospital, they will have done well, and will serve as an example to others.

